# Decisions, Decisions, Decisions: An Ethnographic Study of Researcher Discretion in Practice

**DOI:** 10.1007/s11948-024-00481-5

**Published:** 2024-11-29

**Authors:** Tom van Drimmelen, M. Nienke Slagboom, Ria Reis, Lex M. Bouter, Jenny T. van der Steen

**Affiliations:** 1 Department of Public Health and Primary Care, Leiden University Medical Center, Leiden, The Netherlands; 2 Centre for Science and Technology Studies (CWTS), Leiden University, Leiden, The Netherlands; 3 Amsterdam Institute for Global Health and Development, Amsterdam, The Netherlands; 4 The Children’s Institute, University of Cape Town, Cape Town, South Africa; 5 Department of Epidemiology and Data Science, Amsterdam Public Health research institute, Amsterdam University Medical Centers, Amsterdam, The Netherlands; 6 Department of Philosophy, Faculty of Humanities, Vrije Universiteit Amsterdam, Amsterdam, The Netherlands; 7 Department of Primary and Community Care, Radboud university medical center, Nijmegen, The Netherlands; 8 Radboudumc Alzheimer Center, Nijmegen, The Netherlands

**Keywords:** Researcher discretion, Researcher degrees of freedom, Ethnography, Research integrity, Responsible conduct of research

## Abstract

This paper is a study of the decisions that researchers take during the execution of a research plan: their researcher discretion. Flexible research methods are generally seen as undesirable, and many methodologists urge to eliminate these so-called ‘researcher degrees of freedom’ from the research practice. However, what this looks like in practice is unclear. Based on twelve months of ethnographic fieldwork in two end-of-life research groups in which we observed research practice, conducted interviews, and collected documents, we explore when researchers are required to make decisions, and what these decisions entail.

An abductive analysis of this data showed that researchers are constantly required to further interpret research plans, indicating that there is no clear division between planning and plan execution. This discretion emerges either when a research protocol is underdetermined or overdetermined, in which case they need to operationalise or adapt the plans respectively. In addition, we found that many of these instances of researcher discretion are exercised implicitly. Within the research groups it was occasionally not clear which topic merited an active decision, or which action could retroactively be categorised as one.

Our ethnographic study of research practice suggests that researcher discretion is an integral and inevitable aspect of research practice, as many elements of a research protocol will either need to be further operationalised or adapted during its execution. Moreover, it may be difficult for researchers to identify their own discretion, limiting their effectivity in transparency.

## Introduction

In order to draw attention to the many choices researchers have to make in the process of data collection and analysis Simmons et al. (2011) introduced the concept *researcher degrees of freedom.* These degrees of freedom, also referred to as “researcher initiative” (Glaeser, 2006), or “researcher latitude” (Fletcher & Black, 2007; Humphreys et al., 2013; Mayo, 2018) refer to the discretion that researchers possess in the process of conceiving, planning, executing, and reporting a research project, leading to substantial flexibility in how the research project is conducted.

Scholars studying the topic have generally concluded that this researcher discretion, and the resultant flexibility, is something to be avoided. For example, researchers concluded that flexibility in research is a necessary cause of selective reporting (van der Steen et al., 2018, 2019). John Ioannidis (2005, p. 698) wrote that “[t]he greater the flexibility in designs, definitions, outcomes, and analytical modes in a scientific field, the less likely the research findings are to be true”. And Simmons et al. (2011, p. 1359) themselves added that “flexibility in data collection, analysis, and reporting dramatically increases actual false-positive rates”.

In accounts of how exactly researcher discretion affects research quality, researchers are portrayed as either perpetrators or victims. In the first case, researchers are thought to wilfully and opportunistically use this flexibility to cheat to report results where there are none (Bakker et al., 2020; John et al., 2012; Wicherts et al., 2016). As happens so often though, perpetrators can be victims themselves as well, and researchers can be regarded victims of the current incentive structure in academia that tempts them to ‘cut corners’ (Bouter, 2015; Haven et al., 2019a; Munafò et al., 2020; Nosek et al., 2012; Ware & Munafò, 2015), a defective local research climate (Haven et al., 2019a, b), or simply of their own human nature, tempting them to engage in questionable research practices (DeCoster et al., 2015; Gelman & Loken, 2013; Nuzzo, 2015). Simmons et al. also noted this when they conclude that “people are self-serving in their interpretation of ambiguous information and remarkably adept at reaching justifiable conclusions that mesh with their desires” (Simmons et al., 2011, pp. 1359–1360).

When flexibility is identified as the problem, the solution that follows logically is increased rigour and transparency. Ioannidis for example argued that we should take to heart “the principles of developing and adhering to a protocol” (2005, p. 701). Brian Nosek added a personal note when he said “I have enormous flexibility in how I analyse my data and what I choose to report. This creates a conflict of interest. The only way to avoid this is for me to tie my hands in advance. Precommitment to my analysis and reporting plan mitigates the influence of these cognitive biases” in Nuzzo (2015). Wicherts et al. (2016) continued down this line and proposed that we make research protocols that are “*specific*, *precise*, and *exhaustive* [emphasis in original]”, resulting in a protocol that “describes all steps, with only one interpretation, and excludes all other possible steps”. To aid researchers in drafting such protocols, these researchers delivered an extensive mapping of researcher degrees of freedom (Wicherts et al., 2016).

Though research, both empirical (Fanelli, 2009; John et al., 2012; Martinson et al., 2005) and theoretical (Ioannidis, 2005; Simmons et al., 2011; van der Steen et al., 2019), certainly indicates that researcher discretion can negatively affect the quality of research, an empirical study of this discretion in research practice is missing. Therefore, in this ethnographic research project, we aim to explore when researchers are required to make decisions, and what these decisions entail. This first report outlines the mechanisms through which researcher discretion emerges. This will serve as a foundation for further reports exploring how researchers navigate their discretion, and a mapping of which considerations play a role in these processes. Our ethnographic method is specifically suited for this goal as it lets us “see the relative messiness of practice. It looks behind the official accounts of method (which are often clean and reassuring) to try to understand the often ragged ways in which knowledge is produced in research” (Law, 2004, pp. 18–19).

## Note on Definitions Used

As with many concepts, the concepts of “rigour”, “flexibility”, and “transparency” may be interpreted differently by different people. Popper (1962) taught us that fighting over a definition is about the least productive way of spending one’s time. At the same time, he stressed the importance of specifying what you mean by a concept before explaining your position on it. Throughout this article, we will use a dictionary definition of “rigour”, strictly referring to the quality of rigidity or strictness; of executing a research protocol with none, or a minimum of interpretation. Please note that this is not the only possible definition of the concept, see for example Haven et al. (2022) and Munafò et al. (2014) who employ a broader definition and use “rigour” as somewhat synonymous with research quality in general. We will use “flexibility” as an antonym of “rigour”. Lastly, “transparency” will refer to the quality of making it easy for others to understand what you have done- and which decisions you have taken.

## Methods

### Ethnography

Our research is based on twelve months of ethnographic fieldwork at end-of-life research groups in North-West Europe between November 2020 and February 2022. Ethnographic methods focus on observation of people going about their everyday lives and explore the logic(s), value systems, and contexts that guide social practices (Daynes & Williams, 2018). Participant observation allowed us to witness the everyday decision-making as it happens, and to describe the deliberative processes and the context of researcher discretion in daily practice.

End-of-life care research lends itself well for research on researcher discretion as it may be particularly visible in the discipline, due to its emotionally charged and polarised subject. In addition, randomised controlled trials or other established study designs that are supposed to limit researcher discretion best are not always feasible or ethically acceptable in end-of-life research (Grande & Todd, 2000).

This study is part of a larger ethnographic research project on researcher discretion. Our project protocol was pre-registered on the Open Science Framework (https://osf.io/qmdh5). Our study was deemed not subject to the Dutch Medical Research with Human Subjects Law by the Medical Ethics Review Board Leiden, The Hague, Delft (23-09-2020, #N20.131). Informal methodological advice was provided by the Amsterdam Institute for Social Science Research (AISSR) Ethics Advisory Board.

### Data Collection

The first author (TvD) conducted fieldwork in two research groups. Each period of fieldwork spanned six months, allowing for a degree of immersion necessary for ethnographic observation (Creswell & Miller, 2000; Goffman, 1989). The researcher was embedded fulltime in the participating research groups; he was present during regular working hours, attended professional and social functions with the groups, and sat in on weekly progress meetings. In both research groups, three research projects were selected for detailed observation. In addition, various documents, such as minutes, agendas, and manuscripts, were collected for data analysis. When observational data required further explanation by the participants, the ethnographer asked for additional information directly or scheduled a more or less formal interview. These could range from a lunch meeting to a formal interview lasting between 30 and 75 min. Member checking occurred continuously throughout the research, both as a method to raise validity as well as a courtesy to the participating research group (Cho & Trent, 2006; Koelsch, 2013).

The bulk of the data consists of fieldnotes from meetings. Fieldnotes are detailed descriptions based on heuristic jottings made during the observation (Emerson et al., 2011). At the first research group, 73 meetings in total were observed, at the second research group 45 were observed. In addition, informal conversations with individual group members took place and were documented as additional fieldnotes. We sought triangulation of the findings by supplementing participant observation with document analysis and semi-structured interviews. Interview guides were drafted for each individual interview on the basis of specific observations concerning the individual researcher.

### COVID-19 and Digital Fieldwork

Both stretches of fieldwork took place at different points during the COVID-19 pandemic, rendering traditional ethnographic research based on physical attendance at times impossible. We followed the workflows of the respective groups, which meant that fieldwork at one group took place almost entirely digitally. As a result, a considerable part of the fieldwork consisted of online (semi-)formal group meetings.

### Analysis

Atlas.ti coding software was used in the data analysis. Data analysis was performed by TvD under supervision of [NS, RR, and JvdS], with whom non-recognisable excerpts were discussed.

We performed an abductive analysis, characterised by an iterative cycle between alternating deductive phases in which hypotheses were formed and inductive phases in which these hypotheses were tested; the aim of which was to search for instances which would disprove the hypotheses (Timmermans & Tavory, 2022). First, the data was coded to identify instances of researcher discretion. Grouping coded passages in the data that correspond to the same category of researcher discretion led to iteratively developed axial codes, or categories of researcher discretion. These resulting categories were subsequently analysed to come to the themes presented in this manuscript. Though the open coding of additional projects usually led to different examples of instances of researcher discretion, the last two research projects did not lead to a change or an addition in the final themes. As such, we are confident that we reached inductive saturation on these themes (Saunders et al., 2018).

### Informed Consent, Confidentiality, and Data Management

Research groups were approached through their primary investigator, who after explanation of our research aims and methods inquired whether the rest of the group would be interested in participating. At least one session per group was organised during which we explained our research aims and methods to the entire research group.

All participants were asked to sign an informed consent sheet. This consent sheet can be found in the supplementary materials on our OSF page (https://osf.io/tqwgp/). However, following standard ethnographic practice, consent to participate in this research was ‘fluid’; seen as a process, not an event (Iphofen, 2013). As such, the participants’ consent was renegotiated and reiterated when necessary. Participants were informed that they could withdraw or alter their consent at any point throughout the research, without having to provide a reason.

Because of the potentially sensitive nature of the observations we anonymised names of individuals, institutions and locations (Iphofen, 2013; Walford, 2005). Consequently, we are unable to provide exact demographics of the participating research groups. Both research groups consisted of 15–25 researchers, including senior researchers, postdoctoral researchers, PhD candidates, junior researchers, and research interns. In choosing our fieldwork locations we sought to balance the generalisability and depth of the data. Therefore, the two groups that participated in our research differed in main methodology: one being more qualitatively focused, and the other using quantitative, qualitative, and mixed methods.

To maximise non-recognisability, the following measures were taken. Each participant name was pseudonymised before they entered the ethnographer’s notebook, digital fieldnotes, or transcripts so no real names are present in the primary data. These pseudonyms were chosen with the help of a random name generator to avoid associations to the real name.

However, there may have been cases in which the specificity of the context of the case meant that we were unable to describe the decision accurately and non-recognisable at the same time. In these cases, the researchers followed ethnographic practice and omitted, moved, or changed non-relevant contextual aspects to render the case non-recognisable in publication and presentation (Murphy et al., 2021; Saunders et al., 2015; van den Hoonaard, 2003). For example, if a person’s gender was irrelevant to the research analyses, this may have been changed in this publication to maximise non-recognisability. This implies that the data provided in this publication cannot be used to serve arguments other than the ones central to this manuscript. As an additional check on non-recognisability, representatives of both participating research groups were asked to review the manuscript of this article and deemed it sufficiently non-recognisable for academic publication.

We acknowledge the potential value of providing an open dataset in general (Nosek et al., 2015), including in ethnographic research (Dilger et al., 2019; Murphy et al., 2021). However, because of the abundance of identifying factors in this project’s fieldnotes, we decided not to share this project’s data.

In the findings section we will distinguish two types of quotes. We used double quotation marks to denote direct quotes that were either directly noted down or audio recorded. Single quotation marks denote an interaction reconstructed from the fieldnotes (Murphy et al., 2021).

## Findings

### The Emergence of Researcher Discretion; Under- and Overdetermined Research Protocols

Our coding of the ethnographic data led to a detailed categorisation of when researcher discretion emerged in the research process. The full categorised list of instances of researcher discretion we observed can be found in the supplementary materials on our OSF page (https://osf.io/tqwgp/). This following section contains the results of our analysis of this categorisation. In the section, we will occasionally include an excerpt from this table to give the reader an indication of the breadth of the instances of researcher discretion that we observed.

### Operationalising Underdetermined Aspects in the Research Protocol

We are in a hybrid meeting to discuss the progress of a research project. Two researchers– a senior researcher and a PhD candidate - are on location at the office. Another senior researcher and the ethnographer logged in from home. The agenda for this meeting was sent the day before and its first item is an update on the project recruitment. During this update, one of the senior researchers interjects and notes that they cannot include participants too close to the determined time of the intervention, since they also still have to fill out a questionnaire beforehand. A short silence is followed by the question: “what did we write in the protocol about that?”. The PhD candidate quickly replies: “it simply says: ‘before the intervention’”.

In this case, the research protocol dryly and seemingly straightforwardly prescribed that *all participants fill out the questionnaire before starting the intervention*. With the first intervention session coming up, though, these researchers found themselves discussing what *before starting the intervention* meant in practice. The protocol did not specify *how long before* the intervention the questionnaires had to be completed. One of the researchers noted that following the protocol exactly and interpreting the word “before” to mean any time earlier than the start of the intervention would mean they would be able to accept a participant who hurriedly fills out the questionnaire on their phone just minutes before the start of the intervention, adding that that is likely what she would do if participating in such a study– given her full schedule. She continues: “of course that’s not what should happen”, alluding to the fact that the validity of the data obtained from the questionnaire depends on participants filling them out under similar circumstances. Now, the researchers had to decide how long before the start of the intervention they could still accept filled out questionnaires without affecting the uniformity of the data; “an hour in advance?”. In this case, the researchers chose to tackle the problem by sending the participants ample reminders to fill out the questionnaire, hoping to avoid the problem altogether.

One research phase in which researchers were often required to exercise their discretion in operationalising the research protocol was the inclusion. Specifically, we observed that researchers often had to decide whether an unexpected factor would still allow for inclusion of a potential participant. The various instances in which this came up are outlined in Table 1 below.

**Table 1:** Examples of instances of observed researcher discretion involving underdetermined aspects of the protocol

Deciding whether an unexpected factor (not mentioned in inclusion/exclusion criteria) still allows for inclusion of potential participant
when a participant does not agree to allow their data to be used for future research
when a participant expresses desire to make an adjustment to the intervention which may or may not constitute a considerable difference
when a participant was not recruited completely according to the recruitment protocol
when a potential participant can only participate if the interview is held at a different location, time, or medium, which may or may not constitute a considerable difference
when a potential participant does not want to participate in a specific aspect of the intervention which may or may not constitute a considerable difference
when a potential participant is an acquaintance of one of the researchers
when a participant cannot answer questions but a relative offers to do so

As Table 1 shows, we observed various instances from which researcher discretion emerged in this situation. One example of the last instance from the table below concerns a participant who was discharged from the hospital before he could finish the questionnaire. A researcher explains: ‘I called him, but his partner answered the phone. She said that the patient really only slept at this point. She asked whether she could answer the questions’. A senior researcher notes that this might be possible with factual questions– given this is noted and reported adequately– but, she continues, ‘it gets tricky with questions about attitudes. We know that relatives sometimes think they know, these things, but that they don’t really do’. These are important questions concerning the validity of the data, but the protocol did not prescribe specific answers, and the researchers were required to exercise their discretion.

During our observation of the research practice, we routinely found the researchers in situations as described above: more or less struggling with the operationalisation of ambiguity in the research protocol. In these cases, the research protocol was underdetermined, which led to discretion in its execution. Oftentimes in these situations, with the benefit of hindsight, the researchers in questions kicked themselves. The to-be-operationalised concepts suddenly seemed incomplete; *why did we not prescribe this more explicitly from the start?*

### Adapting Overdetermined Aspects in the Research Protocol

However, researcher discretion not only emerged from underdetermined protocols, but also from overdetermined ones. One such example in our data was when researchers needed to decide whether to add, change, or remove an item from a questionnaire. During our fieldwork this question came up frequently and for various reasons, which are outlined in Table 2 below.

**Table 2:** Examples of instances of observed researcher discretion involving overdetermined aspects of the protocol

Deciding whether to add, change, or remove a measurement item from the data collection protocol
when a specific item is often misunderstood by research participants, potentially leading to inconsistent data
when questionnaire length may deter participation, threatening the feasibility of the research project
when participation may or may not be excessively burdensome to participants
when resource constraints in the research project may or may not require a reduced research load
when data collection may or may not be excessively burdensome for researchers
when an additional measurement could potentially improve the research substantially

In each of these cases, the researchers were forced to make a decision that they did not expect to have to take. And each of these cases came with its own accompanying difficulties. For an example of the first instance– when researchers found that specific item was often misunderstood by research participants, potentially leading to inconsistent data– we find ourselves once again in a hybrid project meeting. One researcher notes that in conducting questionnaires, she finds that participants often struggle to answer a particular item. She adds that the participants’ answers are usually conditional; if A, then yes, if B, then no: ‘which box do I check then?’. A senior researcher notes that this could indeed lead to muddled data, as ‘people may say ‘yes’ and ‘no’ even though they have the same answer’, before she adds: “You see how difficult it is to make a good questionnaire!”. In this case, the researchers decided to maintain the questionnaire item as planned and add a reflection on its potential defects in the “Discussion” section of the research report. However, for the subsequent two instances in the table this would not suffice. If there is a risk that the length of a questionnaire deters participation and as a result the research project may not be finished there is a compelling reason to adapt the questionnaire, as was the case for another project. After extensive debates with the project partners, they agreed on allowing the option of using a shortened questionnaire. Even more compelling a reason to change the questionnaire is when one of its items is likely to excessively burden participants. As one researcher noted that both the physical and emotional burden of participation can be substantial for certain participants. Though most research projects we observed discussed this option, none formally changed their data collection protocol because of this. As such, even though the researchers did not expect these decisions to have to be made, they still had to exercise their discretion.

Researchers working on another project faced a similar situation during their research. Their research protocol specified that they were looking to recruit participants who suffered from a “life-threatening condition”. The criterium of whether someone is suffering from a life-threatening disease seems rather simple. However, these researchers found that occasionally it is not all that clear whether a condition is life-threatening, or that how a condition threatens one’s life can be very different. In one of their project meetings one of the researchers noted that she is finding it difficult to accurately operationalise this inclusion criterium. She explained:

*“The question ‘is it life-threatening?’ is just quite tricky. (…) these heart conditions are so on-and-off. (…) but they’re really one bad operation away from passing away. (…) but you also don’t want to be too active that we end up scaring them”.*

What these researchers effectively found is that their study population might actually be more heterogeneous than expected. One person suffering from a life-threatening condition may live a relatively normal and long life but may suddenly face a flare-up of their condition. Another may be confronted with a maximum life expectancy of two years, of which most will be spent in the hospital. The current inclusion criteria required the researchers to include both patients though their outlooks may be very different. This created a potential difficulty for the research project, since if the researchers accidentally were to include a majority of either patient subgroup, their results may not generalise over their complete study population. As such, they were required to exercise their discretion in adapting the protocol, either by focusing on one patient subgroup, or making sure their participant sample was representative of both.

These were the situations in which only little operationalisation may have been necessary here as clear instructions for action are given– but these actions were no longer possible or desirable under the circumstances at hand.

The instances of researcher discretion in our data can be categorised to emerge under two different circumstances, when the protocol is either under- or overdetermined. When confronted with underdetermined plans, researchers came across passages in the protocol that were not sufficiently specific to prescribe the required actions and needed to be further *operationalised*. These situations were different from moments where the protocol was overdetermined. In these cases, even though the protocol specified a certain action, this action was either unfeasible or undesirable given the circumstances at the time. In these cases, the researchers needed to *adapt* the research protocol. Both types of situations led to discretion that researchers needed to exercise.

### Implicit Researcher Discretion

Occasionally, it was very clear when a situation amounted to a decision by the researchers. However, far from all decisions were so easily identified and many were in fact implicit. Generally, we speak of a decision when different options are contemplated, and one is chosen. But when researchers did not consider other options, that does not mean that these options were not present and they *could* have in fact chosen otherwise, and they did still exercise their discretion, albeit unconsciously.

In fact, this led to a difficulty we encountered in our analysis arising from the question: when is a decision a decision? We set out to map researcher discretion, but what if the researchers did not identify their own degrees of freedom? On occasion, the ethnographer observed actions that could be identified as decisions which the researchers did not identify as such. For example, when one of the junior researchers on a project lamented that it was so awkward listening to the recording of her recent interview because she was confronted with her constantly affirming ‘uhuh’ sounds. The senior researcher responded that that this was no problem, since their transcriber would simply leave them out of the transcript. For the ethnographer, this appeared to be a clear research decision, as the choice to focus on the content of an interview without taking into account the context and affective components of the interaction has far-reaching impact on the outcome of the research project, simply because it constitutes a decision on what is considered *data*. As such, the ethnographer would have classified this as an operationalisation of a simple ‘interviews will be transcribed’ phrase in their protocol and a substantial research decision. However, these researchers did not give it further thought, likely guided by their own research experience and disciplinary background. However, this proved problematic for our own analysis, since a clear-cut identification of researcher discretion proved impossible.

We found that this difficulty of identifying implicit researcher discretion was shared by our participants. As also amongst the participating researchers it was occasionally not clear which topic merited an active decision, or which action could retroactively be categorised as one.

#### “But what Does that Mean?”

Evening is setting and we are in a large conference room at a university, where all research collaborators of a specific research project have come together to discuss the project’s progress. We move to point two on the agenda, about the collection of data on healthcare usage. Irene introduces the topic and describes an unexpected complication they encountered during the study. Occasionally, she explains, a participant may pass away at a different location than where they were originally included into the study. In these cases, they have no easy access to information on how much care the participant received at this other location– be it a hospice, another hospital, or simply their home– though this information is important for one of their research questions. She explains that they agreed with their international research collaborators that they would ask for the healthcare usage at the location where the participant passed away if possible. At this point another one of the researchers, Charlotte, interjects. She notes that she does not really get what the issue is here. ‘It seems pretty clear right?’ she says, that they will ask for healthcare usage at the second site ‘*if possible’*. To which Irene quickly replies: “yes”, she says, “but what does that *mean*?”

In the situation described above, over- and underdetermination of the research protocol converge. The research protocol did not foresee in the participating patients being transferred from the site of inclusion. When the researchers found that they do occasionally move, the protocol needed to be adapted. This first instance of researcher discretion is given to us as an announcement; Irene explains that in a meeting with the collaborators they *decided* to deal with this unforeseen constraint in a particular manner. However, it soon became apparent that this adaptation still needed to be operationalised. The researchers agreed with the collaborators that they would try to gather this data ‘*if possible*’. But as the passage continues, we see that what this actually entails in practice is more difficult to interpret than Charlotte initially recognised.

Everyone now shares their considerations: Maartje, a PhD candidate, responds and says: ‘though admittedly we can’t really do this, but I’ve heard from colleagues who simply use the phone and administer the questionnaire to the doctor of the location where the participant passed away’. Irene says that that will still take half an hour extra per participant at least. Sonja adds that they also have to think about the permission; ‘the doctor in question needs to know that the patient consented to share this data. How did they do that then if they administered the questionnaire over the phone?’. Marit interjects: “they’re supposed to, yeah!”. Emma proposes a solution. She says that they can first email the patients’ consent forms, and then call the doctor in question. Marit promptly remarks that the consent forms can only be sent through secured e-mail.

This discussion continued for a while longer, ostensibly disproving Charlotte’s observation that it seemed ‘pretty clear’ what the researchers should do. Every option that was introduced carried considerable downsides: Irene mentioned the time constraints on the part of the researchers. Sonja and Marit introduced ethical factors. And later in the meeting the time constraints of the health care professionals and a potential discrepancy between different data collection methods also come up.

#### “Which Decisions did We Make Exactly?”

This difficulty of identifying researcher discretion occurred to the researchers themselves as well in fact. On numerous occasions the researchers made references to research decision logs. For example, when a senior researcher explained to a junior researcher that “these are important things for the research log. Because in half a year you’ll have forgotten why we did this,” ‘and then a reviewer comes along, and you can’t explain it anymore’. But this often turned out to be easier said than done as the junior researchers were often unsure of whether a particular action merited inclusion in this research decision log. This difficulty was illustrated by the junior researcher noticing: “How do you actually go at that? What needs to be included? Which decisions did we make exactly?”.

Apparently, researchers’ identification of discretionary space can diverge, making it difficult for both the researchers themselves, as for our study aims to identify when a decision is required or has been made.

## Discussion

### Scientific Rigour and Researcher Discretion

Methodological rigour, defined as the principle of executing a study exactly as one has planned, is often presented as a way of offering more or less *bankable guarantees* for research quality. Viewed from this perspective, the less discretion a researcher possesses the better the research quality (Bakker et al., 2020). Interestingly, the view that good science is inflexible science, is perhaps symptomatic of a larger cultural change concerning discretion. From a historical study of rules and rule following, Lorraine Daston concluded that the general connotation of discretion has changed diametrically in the past centuries. She noted that for someone like Aristotle, it was clear that following a rule or a plan necessarily meant *interpreting* it. In fact, he even listed the faculty of practical judgement, or phrónēsis, as one of his four cardinal virtues. In contrast, Daston concludes that currently, discretion has an explicitly negative connotation, where “exercising discretion in the modern sense stands opposed to executing the rules faithfully” (Daston, 2022, p. 39).

However, our ethnographic study suggests that researcher discretion may be inevitable. Seemingly clear research protocols harbour a great deal of researcher discretion down the road as research plans often require further interpretation. One strategy of avoiding this discretion is to simply aim at writing exhaustive research protocols (Wicherts et al., 2016), However, we observed that this precision in planning can be a source of researcher discretion in its own right; when detailed protocols are no longer implementable or desirable to implement, the plan requires adaptation, and researcher discretion emerges anew. This finding may perhaps be seen as the other side of the coin of recent ‘many analysts’ studies, one of which suggested that “significant variation in the results of analyses of complex data may be difficult to avoid, even by experts with honest intentions” (Silberzahn et al., 2018, p. 338).

Though we show that both under- and overdetermination *can* indeed lead to substantial researcher discretion, our study is not suitable to make any statements about the (im)possibility of drafting a ‘perfectly determined’ or ‘suitably determined’ plan, or what this plan may look like. This could theoretically be done with the use of contingency planning, considering different scenarios (Baldwin et al., 2022; Nosek et al., 2018). However, though this is definitely possible– and indeed recommendable– in certain situations, it is clear that not all contingencies can be planned for. And even in planning for the in theory knowable contingencies in research practice, one is quickly faced with diminishing marginal returns on the time spent planning, for example when this means drafting contingency plans for each questionnaire item if a potential misunderstanding arises in the data collection.

Consequently, research plans must contain an element of vagueness, and researchers must navigate between underdetermination and overdetermination in their research protocols. A similar conclusion was drawn by Lucy Suchman, who posited that every action is influenced by its context. She concluded that “stated in advance plans are necessarily vague, insofar as designed to accommodate the unforeseeable contingencies of actual situations of action” (Suchman, 2007 [1987], p. 31). Simmons et al. (2011) also noted this necessary vagueness in plans when they explain that researcher degrees of freedom stem from the fact that it is “rare, and sometimes impractical for researchers to make all these decisions beforehand” (p. 1359).

As such, discretion in research practice should perhaps not be seen as a weakness or a fault in the scientific method, but rather an integral part of it. In this view, proposed by Feynman (1974) and reiterated by Lakens (2022, ch. 5) the integrity of researchers lies not in compliance to rules or adherence to research protocols, but the ability to apply them wisely. It seems like what these authors are arguing for is a return to valuing Aristotle’s practical judgement in research practice.

Of course, not all instances of researcher discretion will have the same impact on the research outcome, nor are all instances inevitable. A further analysis of the observed instances of researcher discretion is needed to analyse the relative impact of individual instances or (sub)categories of researcher discretion, and the potential measures to address them, in order to guide researchers in responsibly exercising their researcher discretion.

This insight could prove valuable to research integrity education, as little is known yet about what methods of research integrity education work (Hiney, 2015; Katsarov et al., 2022).

### Transparency and Reflexivity

Even the most ardent calls for academic rigour come with a caveat that not everything allows itself to be planned, and considerable discretion of researchers is involved (DeHaven, 2017; Nosek et al., 2018, 2019). These authors emphasised that extensive planning is “a plan, not a prison”, and should still allow change; see also Haven and van Grootel (2019). However, this acknowledgement of the inevitability of researcher discretion seems to always be paired with the requirement that such change be diligently noticed and reported transparently.

This principle that researchers be transparent is based on the assumption that transparency is something researchers are able to do but may decide not to do for several reasons. Researchers with malicious intentions may not want to be transparent because that will unmask their deceptions for example. Lazy researchers would not either, since it will take them too much time. In this conception, transparency serves as a good heuristic indicator to filter out these lazy and malicious researchers. However, our findings indicate that many research decisions are actually made implicitly– as a matter of fact. This finding mirrors theoretical work on implicit behaviour going back at least to Gilbert Ryle’s proposed difference between knowing *that* and knowing *how* (1949). Also Michael Polanyi’s *tacit knowledge* (1966) and Daniel Kahneman’s *system 1 thinking* (2011) come to mind.

The fact that this implicit behaviour also occurs in research projects is theoretically obvious, but seemingly largely ignored when thinking about research practice. Transparency is often presented as a foolproof way to identify researcher discretion where it may not be eliminated. But our findings illustrate that a researcher can only be as transparent as much as they are aware of their own discretion.

This raises the importance of awareness of one’s own discretion as a researcher in a research process, and reflects a call previously made by Gelman and Loken when they pleaded that “researchers can and should be more aware of the choices involved in their data analysis” (2014, pp. 464–465). This is perhaps an aspect of research where more quantitative research methodologies are in a position to learn from qualitative methodology, where this attention to one’s own influence on the research process, often dubbed *reflexivity*, is often seen as an essential part of the method (Braun & Clarke, 2019; Finlay, 1998). Though thoroughly rooted in the qualitative methods, the concept’s practical recommendations are easily translated to quantitative methods, and do not require espousing any specific epistemological foundations. A good starting point for applying the principles of reflexivity to quantitative research is Jamieson et al. (2023), who drafted a “beginners guide to engaging with reflexivity”, containing, for example, prompt questions that quantitative researchers can pose themselves at different stages of the research process to stimulate reflexive engagement.

However, while careful examination of a researchers’ own choices and motivations to conduct research is indispensable for research quality, it is not able to *solve* the issue of discretion– if discretion should even be seen as an issue to be solved. For one, all the abovementioned theorists agree that this implicit discretion is very difficult to make explicit, if not to some extent impossible. This means that it is unlikely that researchers will ever satisfactorily be able to identify their own implicit discretion. Moreover, even when discretion is identified, though introspection can prove a powerful tool to identify biases, it is clearly limited as well. This ‘bias blind spot’ is both empirically well-tested, for example by Hansen et al. (2014) and theoretically well grounded, for example by Thomas Kelly, who argued that the bias blind spot is a logical consequence of our cognition, concluding that “even God could not have made us reliable detectors of our own biases by way of introspection” (Kelly, 2022).

Certain (statistical) researchers have already called for embracing researcher discretion by designing methods that can account for researcher degrees of freedom, instead of trying to eliminate them (Goeman, 2016; White, 2000). Specifically, Edward Glaeser notes that “[t]his requires not just a blanket downward adjustment of statistical significance estimates but more targeted statistical techniques that appropriately adjust across data sets and methodologies for the ability of researchers to impact results” (2006, p. 3).

### Strengths and Weaknesses of this Study

In this vein, we also need to contextualise our own findings by reflecting on our own discretion on the present study. Though none of the participating researchers were acquainted with the ethnographer (TvD), most of them knew the principal investigator of the study (JvdS) either from her work, or even personally as colleagues. This proved very conducive in gaining the trust required for participating in this ethnographic study. As a result, no research group decided against participation, mitigating the chance of sampling biases.

Another important note to be added is that though theoretically we suppose the mechanisms of over- and underdefinition of research protocols outlined in this article apply to all research processes to some extent, this is not an empirical conclusion of this article since we only studied end-of-life care research groups. We hope that our research can be contextualised by studying whether these mechanisms also feature in other research disciplines, and if so, which specific instances of researcher discretion may be discipline-specific or carry specific importance to particular research practices.

Finally, a frequently heard source of doubt on the validity of ethnographic fieldwork is that the ethnographer’s presence at the participating group will cause the participants to behave differently than they would normally. The reasoning goes that participants will self-censor and present themselves in a more favourable light when they know that they are being observed. However, given that ethnography (of science) is a settled and mature field of research, numerous techniques exist to account for, or even harness these effects (Clifford & Marcus, 1986; Daynes & Williams, 2018; Duneier, 2011; Olmos-Vega et al., 2023). First, given the long period of fieldwork (six months per research group), it is unlikely that any participant would have substantially been able to alter their behaviour for the full period of fieldwork. Second, when dealing with these observer effects, the ethnographer must constantly reflect on his position in the group. Of course, where it is relatively easy to ‘blend in’ to an environment in a live meeting, during digital meetings of two researchers plus the ethnographer this meant that the screen would be split halfway between the interlocutor and the observing ethnographer, further drawing attention to the observation. Third, even if participants may have self-censored on occasion, the opposite likely occurred as well, where being observed made the participants more aware of their own discretion. In these cases, the visibility of the ethnographer may have even enriched the data, given that participants may have been induced to voice their considerations more (Monahan & Fisher, 2010). Of course, the strength of this study corresponding to the potential weakness of observer effects is that our ethnographic methods actually allowed us to observe abundant instances of researcher discretion as they occurred, alternatives for which are few and far between.

## Conclusion

Our ethnographic research into research discretion suggests that there is no clear separation between the planning and execution stages of the research. Consequently, we may need to refrain from an unqualified call for maximum rigour in research, and abandon illusions that the ideal protocol “describes all steps, with only one interpretation, and excludes all other possible steps” (Wicherts et al., 2016).

Though this should certainly not be read as an argument *against* rigour, we conclude that the call for scientific rigour must be *qualified* to include when and how this rigour is to be expected of researchers. In fact, research can be both rigorous and flexible at the same time. Certain elements of experimental research must necessarily be rigorous for the results to be valid– and Wicherts and colleagues provided an excellent checklist for this purpose. For components that are flexible, either by design or by necessity, researchers must be trained to exercise their discretion as reflexively and transparently as possible. In the heat of the debates on the reproducibility crisis, an unqualified call for limiting researcher degrees of freedom was understandable. Now, however, we need to find out how to qualify this call. Researcher degrees of freedom may indeed be the soil in which undesirable research practices can grow, but taking away the soil to get rid of the weeds would deprive us of a garden in general.

If we acknowledge that every protocol requires discretion in the execution, we should turn our attention to this discretion. The quality– or integrity– of a researcher is then not defined by how rigorous she can work, but how well she manages the flexibility she possesses.

## Appendix Table Containing all Observed Instances of Researcher Discretion per Research Phase

**Table:** All Observed Instances of Researcher Discretion per Research Phase

**Design**	
1.	**deciding on the members of a guiding panel for the research project**
	when current members do not contribute as agreed upon
	when it is unclear which combination of expertise will be best for the project
	when the current members are likely not diverse enough (gender, profession, age etc.)
2.	**deciding how to phrase a specific item in questionnaire/interview guide**
	when it is unclear what phrasing will yield the best data
	when the current questionnaire does not fit with the intended study population
**Recruitment**	
1.	**deciding whether to stop or reduce recruitment at a particular location**
	when population at a particular location may be too frail to participate in the research
	when recruitment at a particular location may pose an excessive burden on recruitment partners
	when recruitment at a particular location may require too many resources
2.	**deciding whether using a particular method requires expert help**
	when it is unclear whether expert help would outweigh the cost in time and money
3.	**deciding whether accommodation of a recruiting partner to increase participation jeopardises the research**
	when a recruiting partner expresses desire to make an adjustment to the intervention which may or may not constitute a considerable difference
	when a recruiting partner requires full control over recruitment at their location
	when a recruiting partner requires ownership of collected data
4.	**deciding on the specific content and wording of (additional) recruitment materials**
	when drafting a presentation of the project for potential participants
	when drafting additional recruitment materials
	when personalising the recruitment material to a specific potential participant
5.	**deciding whether and how to add a new recruitment strategy or site**
	when recruitment is too slow or stagnant
	when specific population segment is over- or underrepresented in current sample and different from intended sample
6.	**deciding whether and how to adjust or clarify/develop inclusion criteria**
	when included participation may be too frail/vulnerable to participate in the research
	when application of inclusion criteria may have unexpected unwanted side effects
	when inclusion criteria may or may not be incorrectly implemented
7.	**deciding between different options in addressing an absent or unresponsive recruiting partner**
	when a recruiting partner proves unwilling or unable to recruit as planned
**Inclusion**	
1.	**deciding whether an unexpected factor (not mentioned in inclusion/exclusion criteria) still allows for inclusion of potential participant**
	when a participant does not agree to allow their data to be used for future research
	when a participant expresses desire to make an adjustment to the intervention which may or may not constitute a considerable difference
	when a participant was not recruited completely according to the recruitment protocol
	when a potential participant can only participate if the interview is held at a different location, time, or medium, which may or may not constitute a considerable difference
	when a potential participant does not want to participate in a specific aspect of the intervention which may or may not constitute a considerable difference
	when a potential participant is an acquaintance of one of the researchers
	when participant cannot answer questions but a relative offers to do so
2.	**deciding in which segment of sample to categorise a specific participant**
	when specific participant can be interpreted to be eligible for two or more population segments
3.	**deciding whether to include an eligible potential participant**
	when including a particular potential participant may require too much resources
	when there is an indication that participation may prove excessively burdensome
4.	**deciding how to interpret ambiguity in inclusion criteria**
	when it is unclear if potential participants’ medical condition fits inclusion criteria
	when it is unclear whether a potential participant sufficiently masters the language of the data collection
	when it is unclear whether participation will constitute an excessive burden for participant
	when it is unclear whether potential participant is able to give informed consent
**Data collection**	
1.	**deciding whether accommodation of a participant jeopardises the research process**
	when a likely ineligible potential participant expresses desire to participate
	when allowing participants a choice in treatment timing may lead to biased data
	when giving participants too much time to respond may lead to overburdening of the researcher
2.	**deciding whether an item of data can be used for analysis**
	when a software error may have occurred that has changed the data
	when the circumstances of data collection may have introduced bias
	when the materials are incomplete
	when the paper did not find an effect (in a systematic review)
	when the participant did not seem to pay much attention to the questionnaire
	when the participant may not have been completely lucid or may not have understood the questions completely
3.	**deciding whether intervention requires further explanation**
	when the intervention may be implemented incorrectly by the research partners
4.	**deciding whether to add, change, or remove a measurement item from the data collection protocol**
	when specific item is often misunderstood by research participants, potentially leading to inconsistent data
	when questionnaire length may deter participation, threatening the feasibility of the research project
	when participation may or may not be excessively burdensome to participants
	when resource constraints in the research project may or may not require a reduced research load
	when data collection may or may not be excessively burdensome for researchers
	when an additional measurement could potentially improve the research substantially
5.	**deciding whether to continue data collection**
	when participant may not be lucid enough to participate
	when participation may constitute an excessive burden for participant
6.	**deciding how to interpret an ambiguous participant answer**
	when answer allows for two or more interpretations
	when answer contains a concept that is difficult to translate
	when answer is conditional
7.	**deciding how to address error in measurement materials**
	when answer boxes are missing in a questionnaire
	when different versions of the questionnaire have been used in data collection
8.	**deciding who will perform the data collection**
	when determining whether the number of interviewers present may bias the data collection
	when the specific researcher is not completely fluent in the language of data collection
	when there are doubts about the researchers interviewing experience and/or skills
9.	**deciding how to address unexpected constraints in data collection software**
	when it proves impossible to set up necessary reminders
	when the data collection software does not allow for a new type of recruitment
**Analysis**	
1.	**deciding who will perform data analysis**
	when there are doubts about the researcher’s analysis experience and/or skills
2.	**deciding between different statistical tests to continue analysis**
	when exact statistical analysis procedures were not specified before data collection
	when it is unclear which statistical analysis is the current best practice
	when it is unclear which statistical analysis would provide the most informational value
3.	**deciding between different approaches in continuing qualitative analysis**
	when multiple analysis options are possible
4.	**deciding how rigorous an additional check on a qualitative analysis should be**
	when a delay in research progress does not allow for all planned additional checks
**Reporting**	
1.	**deciding how to report specific methodological choices**
	deciding how to report specific methodological choices: when the researchers do not unanimously agree on the motivation behind the choice
2.	**deciding whether a specific piece of information is relevant to the manuscript**
	when having to trim a manuscript down to the maximum wordcount
3.	**deciding whether a research claim is warranted**
	when it is not unequivocal that the current data alone supports a specific conclusion
4.	**deciding on the structure of a publication**
	Undecided
	when trying to retain the reader’s attention
5.	**deciding where to publish research findings**
	it is unclear whether the manuscript merits submission to a ‘top’ journal
6.	**deciding whether a research finding warrants a publication**
	when the informational value of the finding is not unequivocal
	when the research quality is lower than desired
	when time/contract constraints require a research team to cut down on their activities
7.	**deciding whether to present findings at an upcoming conference**
	when it is unsure whether findings will be available at the time of the conference
	when the informational value of the research findings is not (yet) unequivocal
	when the novelty of a finding is not unequivocal
8.	**deciding which name to use for a concept in a publication**
	when a concept in the research data is difficult to translate
	when the preferred concept carries a perhaps undesirable epistemological connotation
	when there is confusion/disagreement concerning the implication of a specific concept
9.	**deciding which reviewers to recommend to a journal upon submission of a manuscript**
	when it is not clear who is most suitable to review the manuscript
10.	**deciding which works to reference in a publication**
	when it is not clear whether a statement requires a reference
	when references in the manuscript need to be trimmed to a journal’s maximum
11.	**deciding who is listed as author in a publication**
	when a researchers’ contribution to the manuscript is not unequivocal
	when one of the authors’ contribution exceeds what was agreed upon
	when one of the authors is unwilling or unable to contribute as agreed upon
	when the research contribution does not easily translate to authorship positions
12.	**deciding on the phrasing of a specific finding in a publication**
	it is unclear how to most accurately describe the finding
**Throughout**	
1.	**deciding whether to discontinue part of the research project**
	when a delay in the research progress does not allow for completion of the complete research project
	when a specific segment of the research population proves considerably more difficult to recruit
2.	**deciding between different options in addressing an absent researcher**
	when a researcher leaves for different employment
	when researcher goes on leave (parental/illness)
3.	**deciding how to adapt design in light of the Covid-19 pandemic**
	when research design requires face-to-face contact
	when the pandemic considerably limited the researchers’ ability to recruit
4.	**deciding how to address error in consent form**
	when consent is insufficient for data sharing
5.	**deciding how to advise an editor concerning a reviewed manuscript**
	when the idea in the manuscript is promising, but it is currently very badly written up
6.	**deciding how to best safely share data with project collaborators**
	when data cannot be shared because of funding issues
	when it is unclear whether the participants specifically consented to sharing this data amongst collaborators
	when national laws forbid data sharing
	when the planned data sharing protocols are no longer feasible
	when there are privacy concerns with the data sharing software
	deciding how to divide research outcomes into publications
7.	**deciding how to interpret ambiguity in protocol**
	when a particular concept requires operationalisation
8.	**deciding how to make best use of limited budget**
9.	**deciding how to make best use of limited (wo)manpower**
10.	**deciding whether a particular researcher needs to develop skills for a particular task in the research project**
	when research software may or may not require specific training
	when time pressure may not allow a specific training
11.	**deciding who is allowed to access the project data**
	when an unexpected potential conflict of interests emerges
12.	**deciding whether a specific decision needs to be included in the research decision log**
	when it is unclear whether the operationalisation of the protocol constitutes a decision
13.	**deciding whether to extend the research project**
14.	**deciding whether to include a collaborating researcher**
	when a researcher may add valuable expertise to the team

## Declarations

### Pre-Registration

Our study protocol was pre-registered on the Open Science Framework (https://osf.io/qmdh5).
